# Identification of susceptibility modules and hub genes of osteoarthritis by WGCNA analysis

**DOI:** 10.3389/fgene.2022.1036156

**Published:** 2022-11-18

**Authors:** Yanchao Wang, Wenjun Zhou, Yan Chen, Dong He, Zhen Qin, Zhao Wang, Song Liu, Lei Zhou, Jianwen Su, Chi Zhang

**Affiliations:** ^1^ Department of Obstetrics and Gynecology, Key Laboratory for Major Obstetric Diseases of Guangdong Province, The Third Affiliated Hospital of Guangzhou Medical University, Guangzhou, China; ^2^ Department of Clinical Medicine, The Third Clinical School of Guangzhou Medical University, Guangzhou, China; ^3^ Department of Orthopedics, The Third Affiliated Hospital of Guangzhou Medical University, Guangzhou, China; ^4^ Department of Joint Surgery, The Third Affiliated Hospital of Guangzhou Medical University, Guangzhou, China; ^5^ Department of Clinical Laboratory Medicine, the Third Affiliated Hospital of Guangzhou Medical University, Guangzhou, China; ^6^ Department of Neurosurgery, Shandong Provincial Hospital Affiliated to Shandong First Medical University, Jinan, China; ^7^ Department of Clinical Laboratory, Affiliated Hospital of Shandong University of Traditional Chinese Medicine, Jinan, China; ^8^ State Key Laboratory of Respiratory Disease, Translational Research Centre of Regenerative Medicine and 3D Printing Technologies of Guangzhou Medical University, The Third Affiliated Hospital of Guangzhou Medical University, Guangzhou, China

**Keywords:** osteoarthritis, WGCNA, pathway analysis, protein–protein interaction, SsGSEA, hub genes

## Abstract

Osteoarthritis (OA) is a major cause of pain, disability, and social burden in the elderly throughout the world. Although many studies focused on the molecular mechanism of OA, its etiology remains unclear. Therefore, more biomarkers need to be explored to help early diagnosis, clinical outcome measurement, and new therapeutic target development. Our study aimed to retrieve the potential hub genes of osteoarthritis (OA) by weighted gene co-expression network analysis (WGCNA) and assess their clinical utility for predicting OA. Here, we integrated WGCNA to identify novel OA susceptibility modules and hub genes. In this study, we first selected 477 and 834 DEGs in the GSE1919 and the GSE55235 databases, respectively, from the Gene Expression Omnibus (GEO) website. Genes with *p*-value<0.05 and | log_2_FC | > 1 were included in our analysis. Then, WGCNA was conducted to build a gene co-expression network, which filtered out the most relevant modules and screened out 23 overlapping WGCNA-derived hub genes. Gene Ontology (GO) and Kyoto Encyclopedia of Genes and Genomes (KEGG) pathway enrichment analyses elucidated that these hub genes were associated with cell adhesion molecules pathway, leukocyte activation, and inflammatory response. In addition, we conducted the protein–protein interaction (PPI) network in 23 hub genes, and the top four upregulated hub genes were sorted out (CD4, SELL, ITGB2, and CD52). Moreover, our nomogram model showed good performance in predicting the risk of OA (C-index = 0.76), and this model proved to be efficient in diagnosis by ROC curves (AUC = 0.789). After that, a single-sample gene set enrichment (ssGSEA) analysis was performed to discover immune cell infiltration in OA. Finally, human primary synoviocytes and immunohistochemistry study of synovial tissues confirmed that those candidate genes were significantly upregulated in the OA groups compared with normal groups. We successfully constructed a co-expression network based on WGCNA and found out that OA-associated susceptibility modules and hub genes, which may provide further insight into the development of pre-symptomatic diagnosis, may contribute to understanding the molecular mechanism study of OA risk genes.

## Introduction

Osteoarthritis (OA), a chronic degenerative joint disease, is the leading cause of disability in the elderly, which aggravates the socioeconomic burden and seriously jeopardizes human health worldwide. With the expanding life expectancy of the aging population, the incidence and prevalence of osteoarthritis also increases (Mandl, 2019; Sacitharan, 2019; Yuan et al., 2020). Though several risk factors associated with osteoarthritis have been well-accepted, including genetic predisposition, gender, age, obesity, and trauma, the pathogenesis of osteoarthritis remains largely unclear (Driban et al., 2020; Allen et al., 2022; Sanchez-Lopez et al., 2022; Sim et al., 2022). Furthermore, numerous studies provide solid evidence that cartilage, subchondral bone, and synovium all play prominent roles in the formation and progression of osteoarthritis (Hu W. et al., 2021; Hu Y. et al., 2021; Allen et al., 2022; Lin et al., 2022; Sanchez-Lopez et al., 2022). To date, a series of clinical and experimental studies have highlighted that osteoarthritis is a systemic low-grade inflammation disease, characterized by the activation of inflammatory factors in synovial fluid and cartilage (Musumeci et al., 2015a; Pereira et al., 2015; Mobasheri and Batt, 2016; Scanzello, 2017; Griffin and Scanzello, 2019; Hu W. et al., 2021; Sanchez-Lopez et al., 2022). During the osteoarthritis process, the innate immune system is activated. Immune cells infiltrate the synovium and produce inflammatory signals and chemokines, and after that, hypertrophic chondrocytes also express proinflammatory mediators and trigger its phenotypic shift to degeneration in a vicious feedback cycle. Proliferating synoviocytes and chondrocytes release proinflammatory products and degradative enzymes that eventually increase cartilage degradation and accelerate disease progression (Hunter and Bierma-Zeinstra, 2019). Practically, inflammation has become recognized as a hallmark throughout all stages of osteoarthritis, from the onset of synovitis, cartilage degeneration, and subchondral bone remodeling osteophyte formation to subchondral sclerosis and cyst formation (Charlier et al., 2019; Hu Y. et al., 2021; Lin et al., 2022; Nakasone et al., 2022; Sanchez-Lopez et al., 2022). Thus, a deeper understanding of inflammatory cell activation is a pivotal step to effectively prevent osteoarthritis progression.

In clinical practice, the traditional diagnosis of osteoarthritis relies on patients’ symptoms, radiography, and magnetic resonance imaging (MRI) evaluation, which have limited value in the detection of early OA and disease intervention (Glyn-Jones et al., 2015; Pereira et al., 2015). In the last 2 decades, with the expansion of proteomics and molecular biology, many scientists have devoted themselves to validating biomarkers for early-diagnosis of OA, intervention, prognostics, and novel therapeutic targets (Glyn-Jones et al., 2015; Han et al., 2021). Many potential biomarkers have been proposed, such as IL-6, IL-8, degradation products of collagen and proteoglycan, and microRNAs (Haraden et al., 2019; Zhang et al., 2020c; Han et al., 2021; Ratneswaran and Kapoor, 2021; Weber et al., 2021; Zhang et al., 2021; Nakasone et al., 2022). However, none of them are sufficient to meet the “BIPED” biomarker classification criteria, which stratify biomarkers as burden of disease(B), investigative(I), prognostic(*p*), efficacy of intervention(E), and diagnostic(D) (van Spil et al., 2010; Musumeci et al., 2015b; Haraden et al., 2019; Batshon et al., 2020; Weber et al., 2021). Remarkably, there are no reliable and satisfactory biomarkers in the study of OA, and its clinical application is a relatively distant prospect (Glyn-Jones et al., 2015). Taken together, it is urgent for scientists to find effective and specific biomarkers for OA, which may facilitate clinical decision-making and early intervention so as to decelerate the subsequently clinical debilitating complications of OA.

Indeed, a variety of bioinformatics softwares and databases have been developed for the identification of disease-related pathways, such as WGCNA (Hao M. L. et al., 2021), KEGG enrichment analysis (Yang et al., 2021; Zhao et al., 2021), and GSEA (Xia et al., 2021; Yang et al., 2021; Zhao et al., 2021; Lu et al., 2022). Weighted correlation network analysis (WGCNA) is a powerful method to find modules related to diseases and explore their pathogenesis (Hao M. L. et al., 2021). Genes of microarray or RNA sequence data are sorted into different modules based on their correlation, and then relating these modules to clinical data can find the correlation between modules and traits (Li et al., 2019; Xia et al., 2021).

In the present study, we identified differentially expressed genes (DEGs) by analyzing the microarray data of the synovial membrane from OA and normal groups. Then, WGCNA was used to identify the most relevant modules with OA, which significantly narrows the range of genes to be screened. Additionally, other comprehensive analyses were used to identify potential hub genes and evaluate immune infiltration levels between OA and normal groups. In brief, we found four hub genes, i.e., CD4, SELL, ITGB2, and CD52 that are potential diagnostic biomarkers which may contribute to the diagnosis of osteoarthritis and provide further insight into the underlying molecular mechanisms for OA risk genes.

## Materials and methods

### Sample collection and ethics approval

Ethics approval (The Ethics Committee of the Third Affiliated Hospital of Guangzhou Medical University 2022-No.018) and written informed consent were obtained for the use of tissue samples (synovial membranes) discarded during surgical treatment in the Department of Joint Surgery, The Third Affiliated Hospital of Guangzhou Medical University from April 2022 to August 2022. Tissue samples of osteoarthritis groups were collected from OA patients undergoing total knee arthroplasty surgery (TKA), while samples for non-osteoarthritis groups were collected from patients undergoing knee arthroscopic surgery for cruciate ligament injuries, meniscus injury, or intra-articular fracture.

### Data download and preprocessing

We initially searched OA expression profile datasets from the Gene Expression Omnibus (GEO) using keyword “osteoarthritis” (https://www.ncbi.nlm.nih.gov/geo/). Microarray datasets of synovial membrane (GSE1919 and GSE55235) and subchondral bone (GSE51588) were obtained from platforms GPL91, GPL96, and GPL13497. Synovial tissues were analyzed in the GSE1919 and GSE55235 datasets, including 15 normal samples and 15 OA samples. Meanwhile, subchondral bone samples were selected in the GSE51588 datasets, including 10 samples from normal donors and 40 from OA patients (Table. 1).

**TABLE 1:** Information of the GEO datatsets.

Dataset	Platform	Manufacturer	Group		Tissue type
			Normal	OA	
GSE1919	GPL91	Affymetrix HG_U95A	5	5	Synovial membrane
GSE55235	GPL96	Affymetrix HG-U133A	10	10	Synovial membrane
GSE51588	GPL13497	Agilent-7026652	10	40	Subchondral bone

### Identification of differentially expressed genes

First, raw data from dataset GSE1919, GSE55235, and GSE51588 were read and preprocessed for batch correction and normalization by using R software (version 3.6.1). Then, the “*limma*” package was conducted for DEG screening. After significance analysis (*p-*value < 0.05 and | log_2_FC | > 1 were used as the selection criteria) of expression levels, volcano plots and DEG expression heatmaps were generated by processing the “*ggplot2*” and the “*pheatmap*” R packages.

## WGCNA (weighted gene co-expression network analysis)

Weighted correlation network analysis (WGCNA) is a systematic biological method used to describe gene association patterns between different samples. It can be used to identify highly synergistic gene sets. Based on the interconnectedness of gene sets and the association between gene sets and phenotypes, it can identify candidate biomarkers (Langfelder and Horvath, 2008). We computed the “*WGCNA*” R package to construct a gene co-expression network of OA. First, a sample-clustering tree was drawn to assess the presence of outliers. Second, the adjacency matrix (AM) was transformed into a topological overlap measure (TOM) matrix (scale free *R* ^2^ = 0.80). Third, the “DynamicTreeCut” method was used to classify genes with similar expression profiles into the same gene modules (the parameters were adjusted to minModuleSize = 60 and deepSplit = 2). Finally, we calculated the correlation between different modules with OA pathogenesis and the most relevant modules were selected as WGCNA-derived hub genes.

## Screening and validation of candidate hub genes for GO and KEGG analyses

KEGG (Kyoto Encyclopedia of Genes and Genomes) is a database resource for systematic analysis of gene functions, linking genomic information with higher-order functional information, especially large-scale molecular datasets generated by high-throughput experimental technologies (Kanehisa and Goto, 2000; Kanehisa et al., 2017). To begin with, the intersection of DEGs and WGCNA-derived hub genes was obtained through Venn diagram by Bioinformatics & Evolutionary Genomics software (http://bioinformatics.psb.ugent.be/webtools/Venn/). Those intersection genes were considered OA pathogenesis-related candidate hub genes. Subsequently, we performed gene ontology (GO) enrichment analysis and KEGG enrichment analysis to help us understand the underlying molecular mechanisms of pathogenesis and progression by means of the “clusterProfiler” R package.

## Construction of protein–protein interaction networks of candidate hub genes

The PPI and molecular interaction networks were predicted and visualized by the STRING online database (http://string-db.org) and the Cytoscape software platform. First, we inputted overlapping genes into the STRING database to screen out and draw PPI networks. Second, Cytoscape software was applied to rank the significant genes in PPI networks.

## Construction of a nomogram model based on hub genes

We constructed a nomogram model to predict the risk of OA using the “rms” package (Iasonos et al., 2008; Park, 2018). The performance of the nomogram model was assessed by calculating Harrell’s concordance index (C-index), which can evaluate the predictive ability of the model (Harrell et al., 1996; Chaudhary et al., 2018; Su et al., 2022). Then, we constructed the receiver operating characteristic (ROC) curve by the “ROCR” package to verify the diagnostic efficacy of candidate markers. The area under the ROC curve (AUC) was used as an indicator of accuracy. We used one criterion to distinguish between excellent accuracy (0.9 ≤ AUC<1), good accuracy (0.8 ≤ AUC<0.9), and non-informative accuracy (AUC = 0.5).

## Single-sample gene set enrichment analysis

In order to explore the role of immune cell infiltration in OA, single-sample gene set enrichment analysis was performed by using the“gsva” R package. Gene Set Variation Analysis (GSVA) is a non-parametric, unsupervised algorithm, which is popular in large-scale genomic studies. GSVA does not need to group samples in advance and can calculate enrichment scores for specific gene sets in each sample (Hänzelmann et al., 2013; Ferreira et al., 2021). We also investigated the differential immunocyte infiltration levels among 22 types of immune cells and analyzed the correlation between the candidate hub genes and immune cell infiltration in OA groups by CIBERSORT analysis, a computational method for quantifying cell fractions from tissue–gene expression profiles (Chen et al., 2018; Kawada et al., 2021).

### Primary cell culture

Human fibroblast-like synoviocytes were isolated from synovial tissues of non-osteoarthritis and osteoarthritis groups.

The collected tissues were digested and incubated first for 2–4 h with 0.5% collagenaseⅡand then for 30 min with 0.05% trypsin. The isolated cells were cultured in DMEM/F12 medium (Gibco, Thermo Fisher) supplemented with 10% fetal bovine serum (Gibco, Thermo Fisher) and 1% of penicillin/streptomycin (Gibco, Thermo Fisher) under standard cell culture conditions (5% CO_2_, 37°C). After 24–48 h, supernatants were discarded in order to remove non-adherent cells without disturbing adherent cells. Human synoviocytes in passages 4 to 6 were collected for subsequent experiments.

### RNA isolation and real-time quantitative PCR analysis

Total RNA of primary synoviocytes was collected according to the instructions of a TRIzol kit (Invitrogen Corporation, Carlsbad, CA, United States), and cDNA was synthesized with the reverse transcription kit (Takara). Real-time quantitative PCR (qPCR) with SYBR Green detection chemistry was performed on a Roche LightCycler480 Ⅱ Real-Time PCR system (Roche Diagnostics GmbH, Forrenstrasse 26,343, Rotkreuz, Switzerland) with the following primers (Table 2). Melt-curve analyses of all real-time quantitative PCR products were performed. All samples were measured thrice, and the mean value was considered for comparative analysis. Quantitative measurements were determined using the △△Ct method, and GAPDH expression was used as the internal control. One-way analysis of variance was used for statistical analysis, and a two-tailed probability (*p*) value < 0.05 was considered statistically significant.

**TABLE 2:** Primers used for RT-qPCR amplification.

Primers	Forward	Reverse
CD4	TGC​CTC​AGT​ATG​CTG​GCT​CT	GAG​ACC​TTT​GCC​TCC​TTG​TTC
SELL	ACC​CAG​AGG​GAC​TTA​TGG​AAC	GCA​GAA​TCT​TCT​AGC​CCT​TTG​C
CD52	TCT​TCC​TCC​TAC​TCA​CCA​TCA​G	CCT​CCG​CTT​ATG​TTG​CTG​GA
ITGB2	AAG​TGA​CGC​TTT​ACC​TGC​GAC	AAG​CAT​GGA​GTA​GGA​GAG​GTC
GAPDH	ACA​ACT​TTG​GTA​TCG​TGG​AAG​G	GCC​ATC​ACG​CCA​CAG​TTT​C

## Immunohistochemistry study

Samples were selected for immunohistochemical staining to experimentally verify the difference in their expression in non-osteoarthritis and osteoarthritis tissues. The tissues were formalin-fixed, paraffin-embedded, and finally cut into slices with a microtome. CD4, SELL, ITGB2, and CD52 were detected using the rabbit anti-CD4 polyclonal antibody (Servicebio, GB11064) at 1:1,000, anti- SELL polyclonal antibody (Affinity Bioscience, Cat#DF6509) at 1:200, anti- ITGB2 polyclonal antibody (Affinity Bioscience, Cat#DF6896) at 1:200, and anti-CD52 polyclonal antibody (Proteintech, Cat No.21809-1-AP) at 1:200. Sections were incubated with the HRP-goat anti-rabbit secondary antibody (Servicebio, GB21303) at a 1:200 dilution for 30 min at room temperature. Color was developed by diaminobenzidine (DAB). The tissue sections were observed under a microscope.

## Results

### Screening of DEGs

**FIGURE 1:**
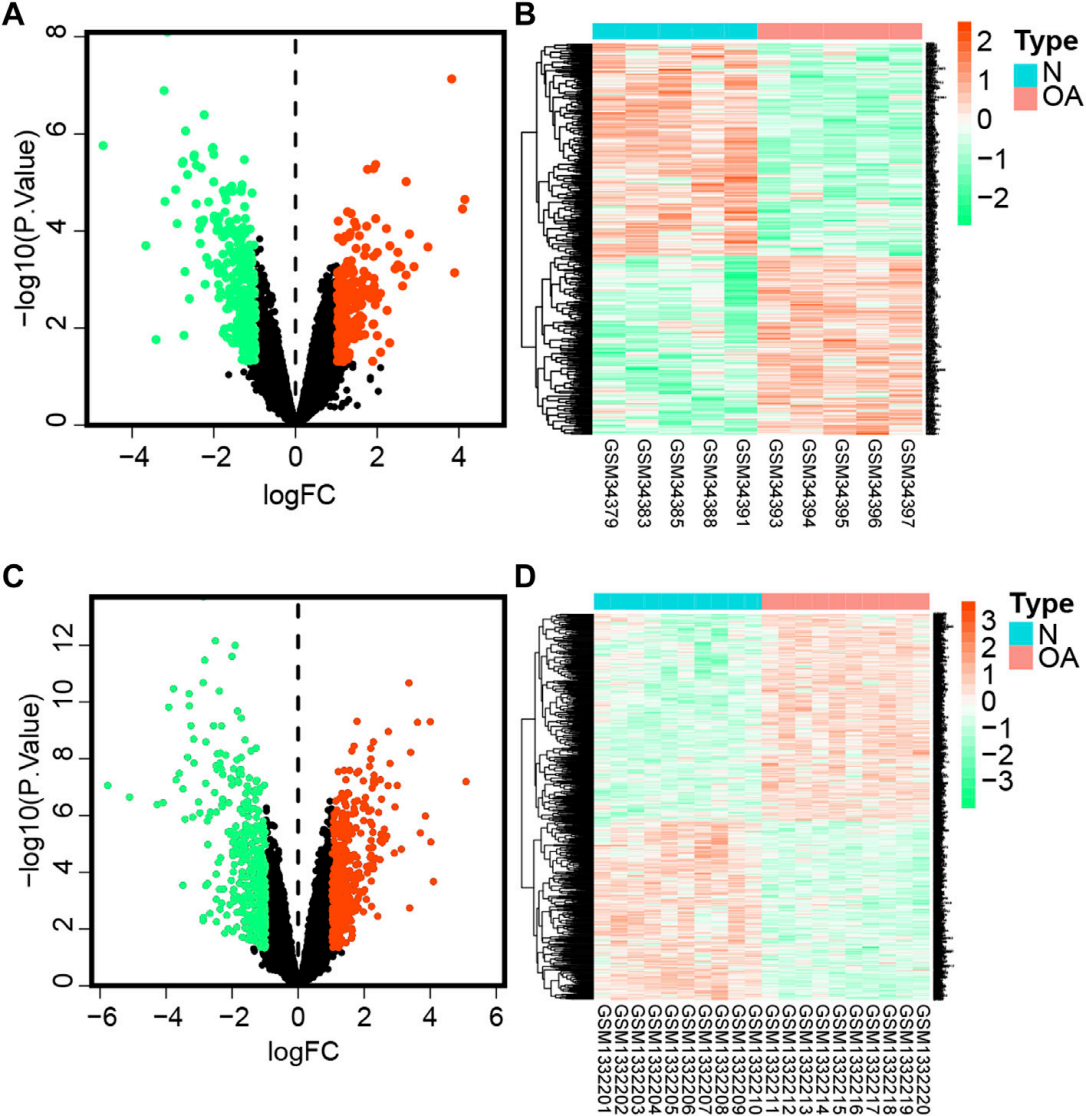
Differentially expressed genes.**(A)** Volcano plot of DEGs in the GSE1919 dataset. The green dot represents downregulated genes, gray represents genes with no significant difference, while the red dot represents upregulated genes comparing the OA group with the normal group in the GSE1919 dataset. **(B)** Heatmap represents hierarchical clustering for DEGs in the GSE1919 dataset. **(C)** Volcano map of DEGs in the GSE55235 dataset. **(D) **Heatmaps of DEGs in the GSE55235 dataset.

After preprocessing raw data, genes with *p*-value < 0.05 and | log_2_FC| > 1 were considered differentially expressed genes (DEGs). In this part, we screened 477 DEGs in the GSE1919 dataset (Figures 1A,B), including 218 upregulated and 259 downregulated genes in the OA group compared with the normal group (Supplementary Table S1). In the meantime, 834 DEGs were observed in the GSE55235 dataset (Figures 1C,D), with 448 upregulated and 386 downregulated DEGs (Supplementary Table S2).

### WGCNA network construction and OA-related module dsl identification

**FIGURE 2:**
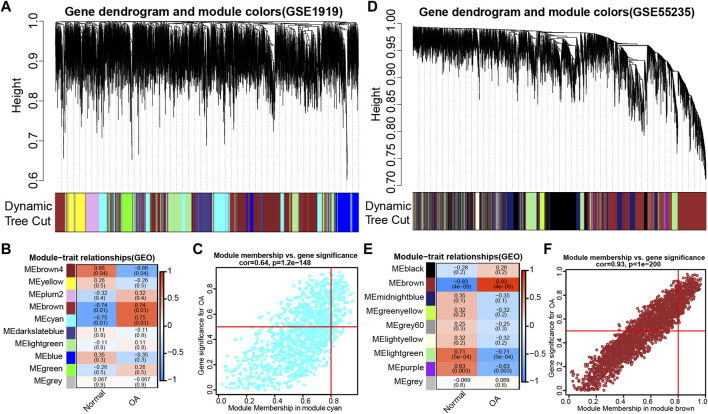
WGCNA was performed to discern osteoarthritis (OA)-related gene modules in the GEO datasets. **(A)** Dendrogram of all genes in the GSE1919 dataset was clustered on the basis of a topological overlap matrix (1-TOM). Each branch in the clustering tree represents a gene, while co-expression modules were constructed in different colors. **(B) **Module-trait heatmap of the correlation between the clustering gene module and OA in the GSE1919 dataset. Each module contains the corresponding correlation coefficient and p value. **(C)** Scatter plot of module cyan has the strongest positive correlation with OA in the GSE1919 dataset. **(D)** Dendrograms of all genes in the GSE55235 dataset were clustered on the basis of topological overlap matrix (1-TOM). **(E)** Module-trait heatmap of correlation between the clustering gene module and OA in the GSE55235 dataset. Each module contains the corresponding correlation coefficient and p value. **(F)** Scatter plot of module brown has the strongest positive correlation with OA in the GSE55235 dataset.

To discern if potential gene modules correlate with OA, we conducted WGCNA to analyze all candidate genes in different stages of osteoarthritis formation from OA-related datasets (GSE1919 and GSE55235) (Figures 2A,D). Based on the average link hierarchical clustering and soft threshold power, we confirmed 10 (Figure 2B) and 9 (Figure 2E) different gene modules on the basis of the GSE1919 and GSE55235 datasets, respectively. After analyzing the positive correlation coefficient, we selected the module with the strongest (and positive) correlation coefficient. Finally, module cyan (r = 0.64, *p* = 1.2e−148) (Figure 2C) and module brown (r = 0.93, p < 1e−200) (Figure 2F) were screened out in the GSE1919 and the GSE55235 datasets, respectively. As a result, 1,283 genes were identified in module cyan of the GSE1919 dataset (Supplementary Table S3), while 2,284 genes were identified in module brown of the GSE55235 dataset (Supplementary Table S4), both of which were selected as WGCNA-derived hub genes.

### GO enrichment and KEGG pathway analyses of candidate hub genes

**FIGURE 3:**
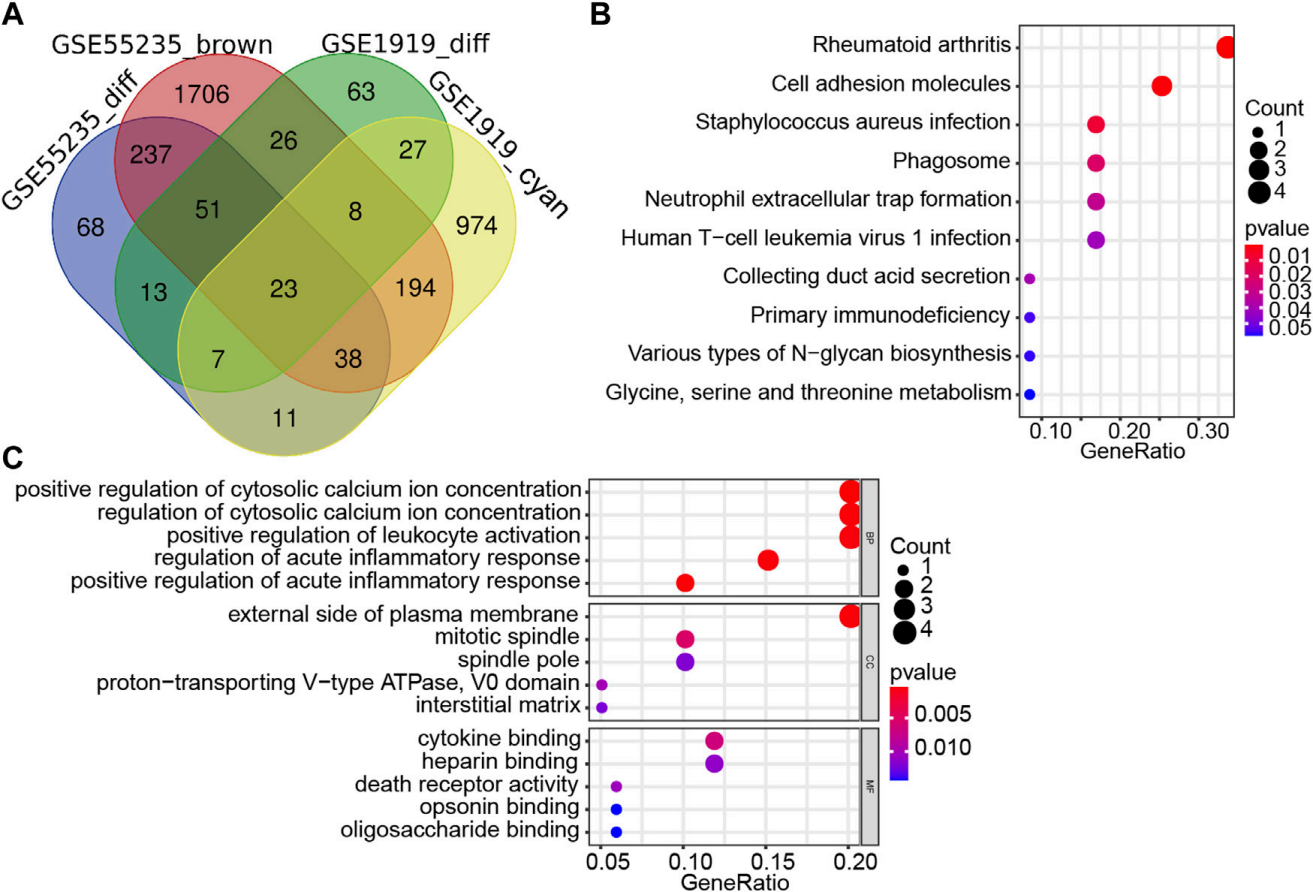
Screening and validation of candidate hub genes. **(A)** Venn diagram revealed 23 overlapping candidate hub genes. **(B)** Kyoto Encyclopedia of Genes and Genomes (KEGG) pathway analysis of candidate hub genes. **(C)** Gene ontology (GO) enrichment analysis of candidate hub genes.

In order to find co-expression genes between DEGs and WGCNA-derived hub genes, we took the intersection of DEGs and WGCNA-derived hub genes by using Venn diagram (Figure 3A). As a result, 23 overlapping genes were screened out as candidate hub genes, which might play a vital role in the formation and progression of osteoarthritis (Supplementary Table S5). To further understand the potential role of those 23 overlapping genes, GO and KEGG analyses were performed. The KEGG pathway analysis showed that these genes were enriched in the rheumatoid arthritis, human T cell leukemia virus 1 infection, and neutrophil extracellular trap formation pathway (Figure 3B), while the GO enrichment analysis revealed that changes in these 23 genes were mainly enriched in the regulation of cytosolic calcium ion concentration, cell adhesion molecules, cytokine binding, leukocyte activation, and acute inflammatory response (Figure 3C).

### Analysis of hub genes through the PPI network

**FIGURE 4:**
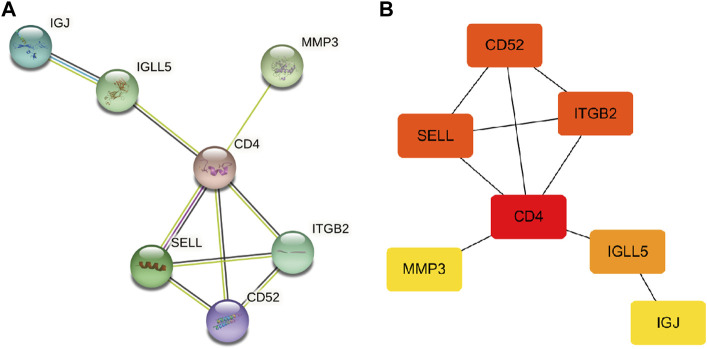
PPI network. **(A)** PPI network of overlapping hub genes. **(B)** Most significant upregulated genes were obtained (the deeper the color, the higher the score).

First, the PPI networks of overlapping hub genes were analyzed by the STRING online tool (Figure 4A). Then, by using Cytoscape software, top four upregulated genes that ranked a high score are visualized as shown in (Figure 4B). Briefly, CD4, SELL, ITGB2, and CD52 were sorted out.

### Nomogram model construction and OA risk prediction

**FIGURE 5:**
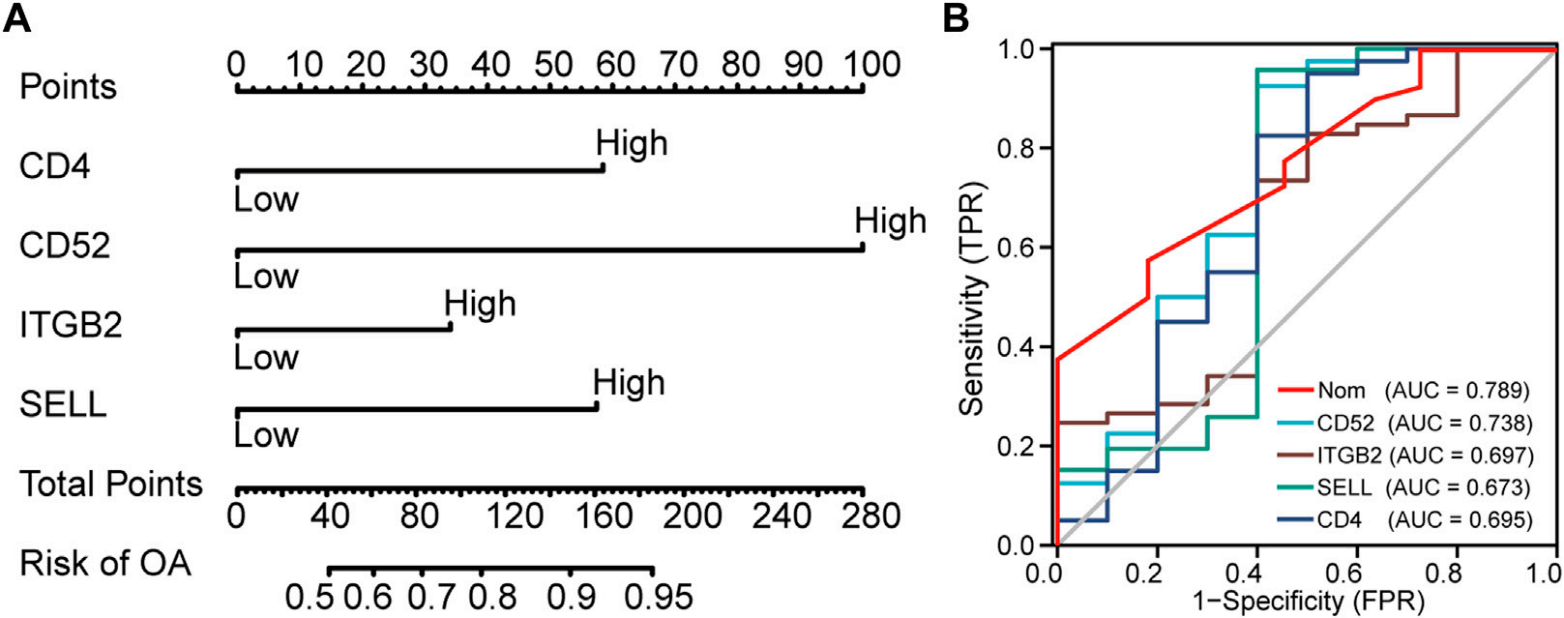
Nomogram to predict the risk of OA. **(A)** Nomogram model of hub genes. **(B)** ROC curves to assess the diagnostic efficacy of our nomogram model and each hub gene.

Subsequently, a nomogram model was constructed to predict the risk of OA (Figure 5A). The discrimination ability of the nomogram was evaluated using concordance index (C-index). The C-index value was 0.76 in our nomogram model. Typically, a C-index value greater than 0.7 suggests a reasonable estimation. As a result, our nomogram model showed an excellent performance on OA prediction. Next, each ROC curve of the four hub genes (CD4, SELL, ITGB2, and CD52) together with our nomogram model was calculated to assess diagnostic efficacy by introducing another external dataset (GSE51588). According to our criteria, 0.9 ≤ AUC<1 represents excellent accuracy, 0.8 ≤ AUC<0.9 represents good accuracy, and AUC = 0.5 represents non-informative accuracy. The area under the curve (AUC) of our nomogram could distinguish OA from the control group. In addition, the diagnostic value was 0.789, higher than that of each hub gene (Figure 5B).

### Evaluation of immune cell infiltration in OA

**FIGURE 6:**
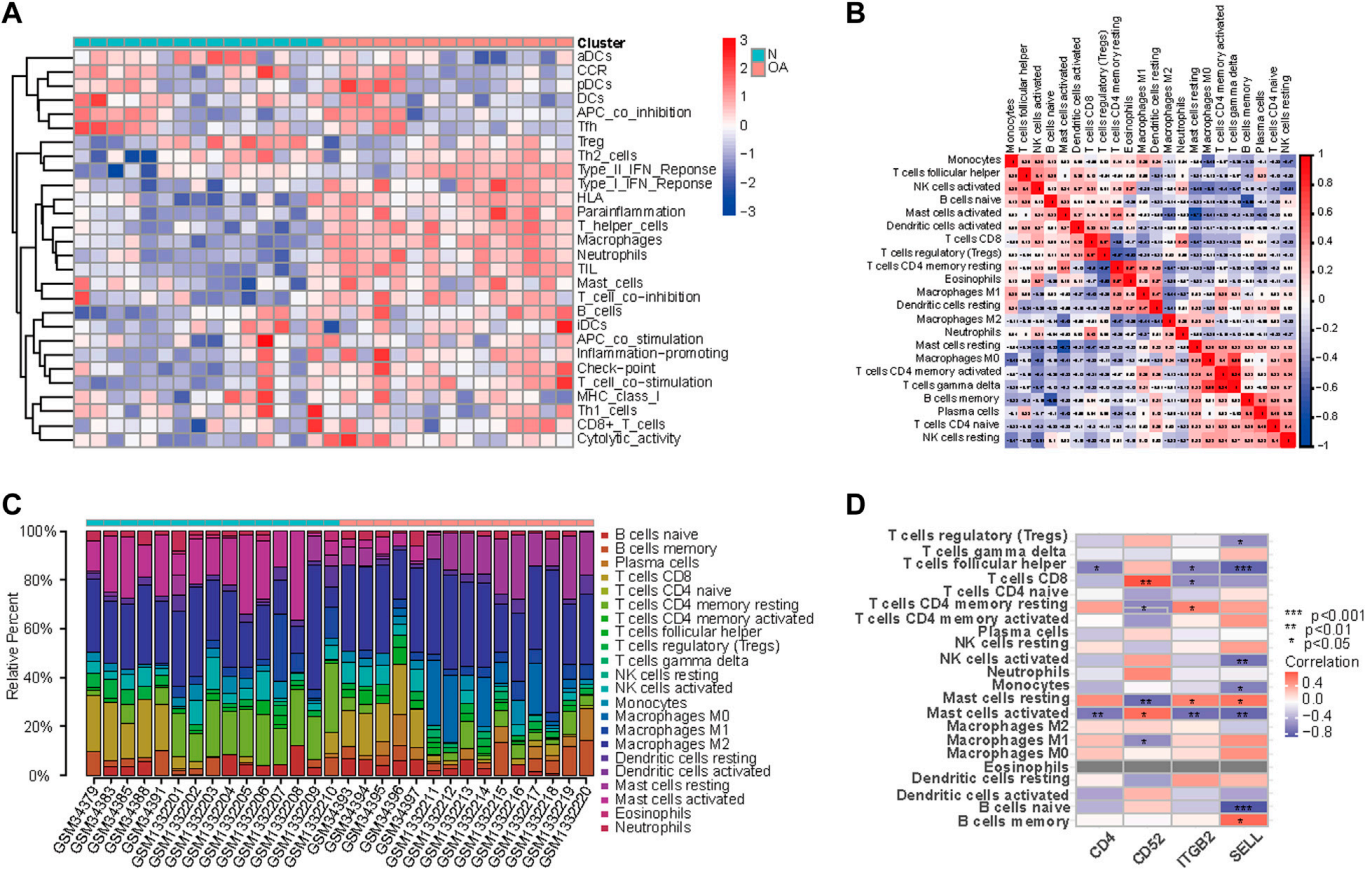
Landscape and correlation heatmap of 29 immune-related gene sets. **(A)** Relative distribution of 22 kinds of immune cells in all samples. **(B)** Correlation heatmap of immune cells in all samples. Red squares indicate positive correlation, and blue squares indicate negative correlation; the deeper color squares indicate stronger correlations. **(C)** Bar chart shows the composition of 22 immune cell types in the two groups. Each color represents a kind of immune cell. **(D)** Correlation between hub gene expression and immune cell infiltration. Red squares indicate positive correlation, and blue squares indicate negative correlation; the deeper color squares indicate stronger correlations.

Since GO enrichment and KEGG pathway analyses revealed that these hub genes were mainly related to inflammation response, the landscape and correlation heatmap of 29 immune-related gene sets were produced by ssGSEA (Figure 6A). The CIBERSORT algorithm was applied to reveal different leukocyte infiltration levels in the two groups. The bar chart shows the composition of 22 immune cell types, while the correlation heatmap revealed different infiltration levels of immune cells in the OA group (Figures 6B, C). Figure 6D illustrated the correlation between hub gene expression and immune cell infiltration. As shown in the heatmap, the expressions of CD4, ITGB2, and SELL were found to be negatively correlated with those of follicular helper CD 4 + T cells and activated mast cells, and the expression of CD52 was found to be positively correlated with that of activated mast cells and CD 8 + T cells. In addition, ITGB2 and SELL expression levels were positively correlated with the resting mast cells. On the contrary, the expression of CD52 was found to be negatively correlated with the resting mast cell infiltration. In the meantime, the expression of SELL was negatively correlated with regulatory T cells (Tregs), activated NK cells, and naive B cells. The expression of ITGB2 was negatively correlated with CD 8 + T cell infiltration, while it was positively correlated with resting memory CD 4 + T cell infiltration.

### Expression of hub genes in clinical specimens of synovial tissues

**FIGURE 7:**
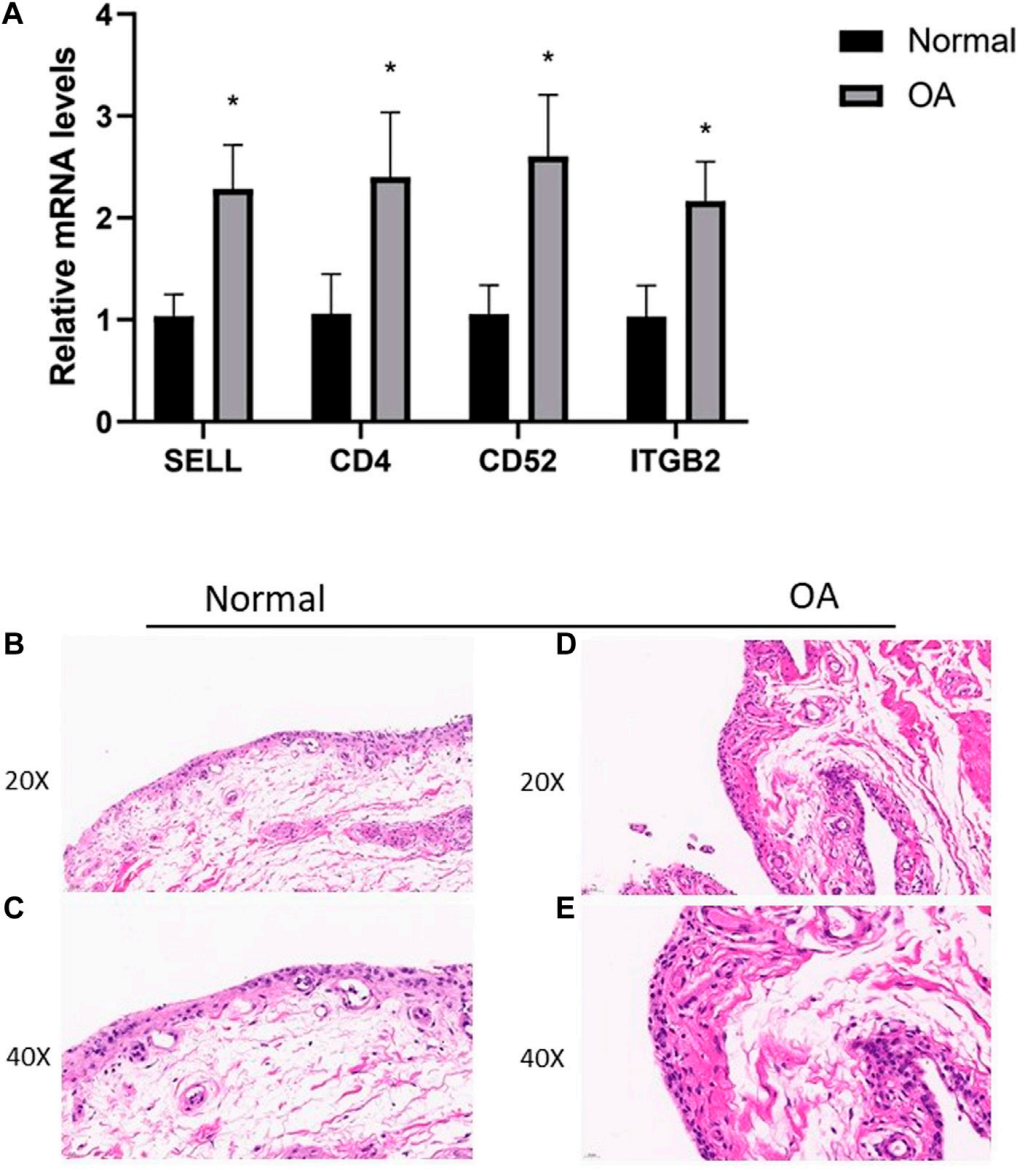
**(A)** RT-PCR validation of hub genes in human primary culture fibroblast-like synoviocytes between OA and normal controls. All experiments were performed thrice, and results were presented as M ± SD. (∗p < 0:05). **(B–E)** HE staining of synovial tissues from TKA patients and patients undergoing knee arthroscopic surgery.

To further verify the expression results obtained by our bioinformatics research, we performed Q-PCR in human primary culture synoviocytes. The results showed that the relative expression levels of CD4, SELL, ITGB2, and CD52 were consistent with our hypothesis (Figure 7).

**FIGURE 8:**
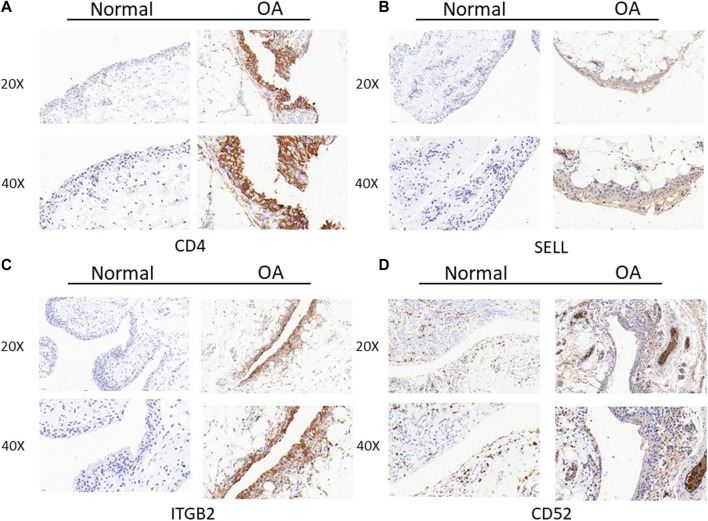
Expression of CD4, SELL, ITGB2, and CD52 proteins in clinical specimens of synovial tissues from TKA patients and patients undergoing knee arthroscopic surgery. **(A) **CD4, **(B) **SELL, **(C)** ITGB2, and **(D) **CD52.

Then, we assessed the expression of CD4, SELL, ITGB2, and CD52 proteins in synovial tissues from TKA patients and patients undergoing knee arthroscopic surgery using IHC staining. As expected, the expressions of CD4, SELL, ITGB2, and CD52 were significantly upregulated in the OA group compared with the normal group (Figure 8).

## Discussion

Osteoarthritis (OA) is the most prevalent and disabling bone disease, with over 250 million people getting affected in populations (Hunter and Bierma-Zeinstra, 2019). In line with the aging population, the absolute number of osteoarthritis cases is currently increasing. OA is a heterogeneous disease with a wide range of underlying pathways, and its pathogenesis is complex and remains largely enigmatic. Even worse, there are no effective biomarkers for early diagnosis of OA.

Diagnostic biomarkers aim to identify patients with pathological changes (Glyn-Jones et al., 2015). Currently, researchers have concentrated on validating the biomarkers involved in cartilage degeneration in OA (Musumeci et al., 2015b; Batshon et al., 2020). Certain molecules such as nitric oxide (NO) and sodium nitroprusside (SNP) have been recognized as biomarkers that are involved in chondrocyte apoptosis and cartilage extracellular matrix (ECM) destruction; however, no deeper analysis of this hypothesized association was described (Musumeci et al., 2015b; Huang L. W. et al., 2021; Roy et al., 2021). The death receptor Fas/FasL, toll-like receptors 1/2 (TLR1/2), collagen markers (collagen type II and type X), and thrombospondin-4 (TSP-4) were also proposed as biomarkers of cartilage degeneration in OA, although these findings lack *in vivo* experiments (Tu et al., 2013; Luo et al., 2018; He et al., 2019; Maly et al., 2019; Barreto et al., 2020). It is reported that interleukin-6 (IL-6), monocyte chemoattractant protein 1 (MCP-1), fibroblast growth factor (FGF-2), and transforming growth factor beta 1 (TGFβ-1) showed a predominant increase in protein levels, while activin A mainly decreased in the synovial fluid of OA patients during knee joint distraction (Watt et al., 2020; Henrotin, 2022). Interestingly, in a study of knee OA, researchers found that serum lipopolysaccharide-binding protein (LBP), IL-6, interleukin-8 (IL-8), tumor necrosis factor alpha (TNF-α), and the cluster of differentiation 14 (CD14) of synovial fluid were associated with most MRI features in an earlier stage of knee OA (Rajandran et al., 2020). Batshon et al. (2020) found that the NT/CT deacetylase sirtuin-1 (SIRT1) ratio correlated with OA in both mice and humans, and this increase was mainly due to NT fragment level increase from non-senescent chondrocyte apoptosis. A phase III clinical trial study proposed that urine CTX-II was associated with risk of radiographic progression, and this biomarker can independently contribute to prediction of structural progression risk and joint replacement surgery (Bihlet et al., 2020). It should be noted that none of these biomarkers is sufficient for use in clinical practice and many obstacles remain. Therefore, more in-depth research studies are needed to identify specific biomarkers that might be beneficial to early diagnosis, intervention, and etiology study of OA (Hunter and Bierma-Zeinstra, 2019; Batshon et al., 2020; Weber et al., 2021). In our study, we screened out four potential biomarkers by comprehensive bioinformatics methods together with molecular biotechniques, computing the diagnostic efficacy of these candidate biomarkers by ROC curves (AUC = 0.789), which proved to be efficient in diagnosis. Nevertheless, little attention has been focused on these candidate biomarkers (CD4, SELL, ITGB2, and CD52) in the progression of OA.

Currently, with the rapid development of computational algorithms and gene chip technology, many researchers have focused on exploring biomarkers of numerous diseases by application of multiple bioinformatic approaches, which enables in-depth analysis of the whole genome and accelerates the progression of disease study. However, many studies are confronted with limited databases (Gao et al., 2019; Cao et al., 2021; Qin et al., 2021), lack of experimental validation (Gao et al., 2019; Zhang et al., 2020b; Cao et al., 2021), controversial methodology validation (Qin et al., 2021; Li et al., 2022), and unconvincing outcomes. Qin et al. (2021) chose only one dataset (GSE55235) for WGCNA analysis and validated their findings in chondrocytes stimulated by IL-1β. First, in terms of precision, their limited dataset may lead to sampling error, and the conclusions may cause bias. Second, experimental validation of the chondrocytes stimulated by pro-inflammatory cytokine could not represent the real condition of OA *in vivo*. Third, adding IL-1β cytokine in the OA group means introducing a new variable which may have adverse impacts on the experiment result. Researchers performed WGCNA analysis to find the miRNA–mRNA network. BTG2, ABL2, and VEGFA were identified and validated in the IL-1β-induced OA chondrocytes (Li et al., 2022). Other research groups also only chose one dataset for WGCNA analysis, and many of them lacked validation experiments (Gao et al., 2019; Zhang et al., 2020b; Cao et al., 2021; Wang et al., 2022) or lacked synoviocyte validation data (Qin et al., 2021; Li et al., 2022). None of them verified and evaluated the diagnostic efficacy of candidate markers by mathematical models. Consequently, these controversial issues suggest that more comprehensive and powerful design should be taken into consideration when we utilize these bioinformatic approaches to the study of diseases.

In this study, we take advantage of the comprehensive bioinformatic analysis to further explore potential hub genes and their interactions with immune cell infiltration that might influence OA progression. We downloaded three mRNA datasets from the GEO database. First, we screened out 477 and 834 DEGs in the GSE1919 and the GSE55235 datasets, respectively. Second, we conducted WGCNA to analyze the most relevant modules in the OA group and screened out WGCNA-derived hub genes. After taking the intersection of DEGs and WGCNA-derived hub genes by a Venn diagram, 23 overlapping genes were found out. Third, GO enrichment and KEGG enrichment analyses were conducted, elucidating that cell adhesion molecules pathway, leukocyte activation, and inflammatory response are essential in OA. Subsequently, we analyzed 23 potential hub genes through a protein–protein interaction network, and the top four upregulated hub genes (CD4, SELL, ITGB2, and CD52) were finally sorted out. Our nomogram model showed good performance in predicting the risk of OA (C-index = 0.76). After that, by introducing another external dataset (GSE51588), we assessed the diagnostic efficacy of our nomogram model by ROC curves (AUC = 0.789), which proved to be efficient in diagnosis. Since GO and KEGG pathway analyses revealed that these hub genes were mainly related to inflammatory response, we took advantage of the single-sample gene set enrichment analysis to discover immune cell infiltration in the OA group. In the meantime, the mRNA expression levels of human primary cultured synoviocytes and immunohistochemistry study of synovial tissues confirmed that the relative expression levels of CD4, SELL, ITGB2, and CD52 were significantly upregulated in the OA group compared with the normal group. The discrepancy between the mRNA and protein levels of the proposed biomarkers could be attributed to the post-transcriptional regulation, such as protein methylation, phosphorylation, and acylation.

To our knowledge, this study for the first time integrates the WGCNA-derived hub genes with differentially expressed genes (DEGs) based on two GEO databases. We first validated our conclusion in human primary cultured fibroblast-like synoviocytes isolated from clinical samples and fresh human synovial tissues. In addition, we first constructed a nomogram model to predict OA risk by using our WGCNA-derived hub genes. Third, we verified the diagnostic efficacy of our nomogram model by receiver operating characteristic curves. Last but not least, rather than verify it in internal datasets itself in many other studies (Huang R. Z. et al., 2021), we introduced another external dataset (GSE51588) to verify the diagnostic efficacy by ROC curves (AUC = 0.789), which certainly can produce more reliable results. Hence, our study may provide a solid foundation and practical design for the molecular mechanism and biomarker study of OA.

Based on the GO database, 23 hub genes were mainly enriched in leukocyte activation, cell adhesion molecules, and acute inflammatory response. These results demonstrated that OA was characterized by immune cell inflammation, adhesion, and infiltration. To be honest, substantial evidence indicated that inflammation is the fundamental pathogenesis of osteoarthritis (Geyer and Schönfeld, 2018; Molnar et al., 2021).

Consequently, many new therapeutic strategies have been proposed and tested focusing on immune regulation by inhibiting inflammation response (McAlindon et al., 2017; Geyer and Schönfeld, 2018; Chen et al., 2019; Zhao et al., 2019; Zhu et al., 2022). KEGG signal pathway enrichment analysis suggested that these genes were enriched in the rheumatoid arthritis, human T cell leukemia virus 1 infection, and neutrophil extracellular trap formation pathway.

In the present study, we found that CD4 is increased in OA tissues. CD4 molecules were extensively expressed in the membranes of many immune cells, such as T lymphocytes, B cells, macrophages, and granulocytes. Much evidence indicated that the levels of soluble CD4 in synovial fluids and sera were higher in OA patients than in healthy individuals (Symons et al., 1991; Sawada et al., 1994; Li et al., 2017). In addition, macrophage infiltration is common in OA synovium, and the predominant cellular infiltrate in OA is macrophage, many of which were CD4-positive (Symons et al., 1991; Zhang H. et al., 2020; Butterfield et al., 2021). Apart from the macrophage, T cells were the second-highest frequency immune cells in the OA synovium and synovial fluid. Accumulating evidence found that CD4+T cells were predominant among T-cell infiltration, and CD4+/CD8+ ratios increased in OA compared to healthy synovium (Dolganiuc et al., 1999; Pawłowska et al., 2009; Lopes et al., 2017; Xia et al., 2017).

SELL (L-selectin, CD62L) is a type-I transmembrane glycoprotein and cell adhesion molecule that is expressed in most circulating leukocytes and regulates leukocyte trafficking to sites of inflammation (Wedepohl et al., 2012; Ivetic, 2018; Ivetic et al., 2019). To date, a series of clinical and experimental studies have highlighted that L-selectin expressed on monocyte protrusion and participated in trans-endothelial migration (TEM). Stimulated by numerous proinflammatory signals, L-selectin is rapidly cleaved from leukocytes and turned over at the plasma membrane through ectodomain shedding, which releases soluble circulating fragment. Therefore, soluble L-selectin was used as a biomarker for leukocyte activity triggered during inflammation (Albertini et al., 1999; Shimada et al., 1999; Font et al., 2000; Giannitsis et al., 2000; Kretowski et al., 2000; Ivetic et al., 2019). Consistent with these studies, our result found that SELL was upregulated in OA tissues compared with control groups in mRNA and protein level. However, scientists observed that SELL was not upregulated in rheumatoid arthritis (RA) synovial tissues, synovial fluid (SF), and peripheral blood (PB). Therefore, their result suggested that L-selectin shedding was not a strict prerequisite for leukocyte migration into synovial fluids and might be mediated in a different pathogenic mechanism in rheumatic diseases (Johnson et al., 1993; Lindsley et al., 1993; Humbría et al., 1994; Björkman et al., 2019).

Integrin β2 (ITGB2), also known as CD18, is a key member of the integrin family which plays a prominent role in the immune system by regulating leukocyte recruitment, aggregation, adhesion, and transmigration during inflammatory diseases. The integrin β2 family consists of different *a*-subunits (CD11a, CD11b, CD11c, and CD11d) and a conserved *ß*-subunit (CD18). Once activated, the *a*-subunits of ITGB2 bind to intercellular adhesion molecules (ICAMs) and promote leukocytes adhered to the endothelial cells in an ITGB2-dependent manner (Hynes, 2002; Ivetic, 2018). However, ITGB2 or CD18 has received little attention in the study of osteoarthritis. Previous studies found that CD18 levels of the synovial membrane were significantly upregulated in rheumatoid arthritis and spondylarthritis (el-Gabalawy et al., 1996; Gjelstrup et al., 2010). Blocking antibodies to CD18 showed inhibition of PBMC binding to synoviocyte monolayers (Lindsley et al., 1993). Interestingly, extensive studies demonstrated that SELL could also activate integrinβ2, promoting adhesion to vascular cell adhesion molecule-1 (VCAM-1) and ICAM-1 (Ivetic, 2018). But even so, the detailed relation of ITGB2 with OA remains poorly defined.

CD52 is widely present on most human lymphocytes and expresses on the cell membrane *via* a glycosylphosphatidylinositol (GPI) anchor (Pemmari et al., 2018). Although little is known about its exact physiological function, CD52 has shown to be a promising target for several immune system-mediated diseases, such as multiple sclerosis (MS), autoimmune inflammatory neurodegenerative diseases, allergic asthma, and lymphocytic leukemia (Shafiei-Jahani et al., 2021). Treatment with CD52 antibody reduced the infiltration of T lymphocytes and macrophages in the spinal cord in MS patients (Hao W. et al., 2021). Scientists established that CD52 is constitutively expressed in innate lymphoid cells under steady state and inflammatory conditions. Anti-CD52 therapeutics ameliorated allergic airway hyperreactivity and lung inflammation (Shafiei-Jahani et al., 2021). However, the role of CD52 in osteoarthritis is explored to a much lesser extent. Further work is needed before a definitive conclusion on this matter can be drawn.

GSEA enrichment analysis revealed comprehensive infiltration levels of immune cells in OA. We found that an increased infiltration of monocytes, follicular helper CD 4 + T cells, CD 8 + T cells, activated NK cells, naive B cells, activated mast cells, and regulatory T cells and a decreased infiltration of resting mast cells, M0 macrophages, activated CD 4 + memory T cells, memory B cells, and naive CD 4 + T cells may be related to the development of OA. To study infiltrating immune cells in knee osteoarthritis, scientists found that CD14^+^ macrophages were the predominant cells in the synovial membrane, followed by CD4^+^ T lymphocytes, whereas mast cells, B cells, and plasma cells were also found, but to a lesser extent (de Lange-Brokaar et al., 2012; Deligne et al., 2015; Moradi et al., 2015; de Lange-Brokaar et al., 2016; Klein-Wieringa et al., 2016; Lopes et al., 2017; Mathiessen and Conaghan, 2017; Scanzello, 2017). The accumulation of macrophages in OA synovium resulted in releasing proinflammatory cytokines including IL-1β and TNF-α, which accelerated cartilage breakdown (Haseeb and Haqqi, 2013). Activated macrophages may develop in response to pathogen-associated molecular patterns (PAMPs) and endogenous DAMPS (Lopes et al., 2017). Although the pathological role of T cells in OA is still unexplained, T cells are the second abundant inflammatory cells in OA synovium (Pessler et al., 2008). Extensive studies demonstrated that CD4+/CD8+ T cell ratios and CD4+T cell infiltration levels have increased in the synovial tissue and peripheral blood of OA patients, indicating that OA is associated with alterations in T cells locally and peripherally within the joint (Hussein et al., 2008; Pawłowska et al., 2009; de Lange-Brokaar et al., 2012; Li et al., 2017). Shan et al. (2017) found that CD4^+^ follicular helper T cells were increased in OA patients and the percent of follicular helper T cells increased with OA grade. Kummer et al. (1994) assessed the expression of granzymes A and B in synovial biopsies of OA and found that NK cells may play a role in the pathogenesis of OA. It was discovered that NK cells are a principal leukocyte infiltrate in the synovial tissue from OA patients. However, with a non-cytotoxic, quiescent phenotype, those synovial tissue-infiltrating NK cells were functionally distinct from blood NK cells (Huss et al., 2010). It is of note that T regulatory cells (Tregs) exert their anti-inflammatory function in the immune system. Nevertheless, little attention has been given to Tregs in the OA progression. In 2014, scientists first found that Tregs were enriched into the joint of OA and RA patients, with CD4^+^CD25+/^high^CD127−/^low^ Tregs enrichment in the peripheral blood and synovial fluid (Moradi et al., 2014; Lopes et al., 2017). Li et al. (2016) found significantly elevated frequencies of CD4^+^CD25^+^Foxp3^+^ Tregs in OA patients. Xia et al. (2017) suggested that LAG-3^+^ Treg cells in OA appeared to reduce their capacity and promote inflammation.

The relationship between hub gene expression and immune cell infiltration was also analyzed. The results showed that the expressions of CD4, ITGB2, and SELL were found to be negatively correlated with those of follicular helper CD 4 + T cells and activated mast cells, while the expression of CD52 was found to be positively correlated with that of the activated mast cells and CD 8 + T cells. Meanwhile, the expression of ITGB2 was negatively correlated with that of CD 8 + T cells, while it was positively correlated with resting memory CD 4 + T cell infiltration. In the meantime, the expression of SELL was negatively correlated with that of regulatory T cells (Tregs), activated NK cells, and naive B cells. However, the underlying mechanism maintaining the relationship between hub gene expression and each immune cell remains to be understood. Meanwhile, q-PCR results of clinical samples showed that four genes (CD4, SELL, ITGB2, and CD52) were upregulated in human primary synoviocytes compared with non-osteoarthritis samples. Furthermore, immunohistochemistry study of synovial tissues confirmed that the expression of these genes in OA tissues was higher than that observed in non-osteoarthritis tissues.

Although these are interesting findings, our study still has some limitations and disadvantages that are worth considering. First, OA is a multifactorial disease, and factors such as genetic predisposition, gender, age, and environmental factors may exert an impact on our result (Driban et al., 2020; Allen et al., 2022; Sim et al., 2022). Second, as the number of publicly available datasets was relatively small, more *in vivo* and animal experiments are needed to confirm the possibilities of our hypothesis. Third, in terms of precision and depth, we strongly agree that the inclusion of additional datasets and next-generation sequencing data would be beneficial for our study analysis. In the early stage of this study, we pre-applied a large number of GEO datasets for analysis, including recent sequencing datasets. However, due to lack of differentially expressed genes (DEGs) or no strong correlation modules screen out by WGCNA, we found certain datasets that could not meet our inclusion criteria and were not suitable for our WGCNA study, such as GSE51588, GSE55457, and GSE117999 datasets. Therefore, the GSE 1919, GSE55235, and GSE51588 datasets were finally chosen in this study.

In summary, our results identified four hub genes including CD4, SELL, ITGB2, and CD52 that may involved in OA progression. Our analyses can provide a solid foundation for better understanding the underlying molecular mechanisms of OA pathogenesis and progression, which may provide more precise and reliable results for pre-symptomatic diagnosis. Further investigations are needed to confirm the possibilities of our conclusions.

## Data Availability

The datasets presented in this study can be found in online repositories. The names of the repository/repositories and accession number(s) can be found below: https://www.ncbi.nlm.nih.gov/, GSE1919; https://www.ncbi.nlm.nih.gov/, GSE55235; https://www.ncbi.nlm.nih.gov/, GSE51588.
